# 

**Published:** 1908-03-21

**Authors:** 


### THE PRICE OF COCAINE

The most remarkable feature of the drug market, at the time of writing, is the phenomenally low price of cocaine. Until the end of last month there existed an agreement between makers which had the effect of keeping the value of this precious alkaloid at a figure which yielded a working profit to the manufacturer, but no sooner had the convention ceased to exist than makers began to compete against each other in a most reckless fashion. The result is that to-day cocaine hydrochloride can be freely procured at a price considerably below the cost of production, contract quantities being quoted 6s. per oz. There can be little doubt that the present is the time to buy, for it seems hardly possible that a lower figure can be quoted, while the price may advance at any moment. It is interesting to note that when cocaine was first introduced into this country, little more than twenty years ago, the price was 1s. per grain.

**A New Medical Directory.**

James Nisbet & Co., Limited, have recently published the first issue of their Medical Directory, which has been in preparation for some time past. We think that it justifies the claim made on its behalf—that it satisfies a long-felt want. It is moderate in size, moderate in price, and remarkably light in weight. The arrangement is novel, and will probably give satisfaction in many quarters. The entire profession, irrespective of broad distinctions of locality, is arranged in alphabetical order, thus facilitating reference. The information attached to each entry is of the shortest, one past appointment and one publication alone being given, but the details appear to be accurate, and the plan of putting the earliest qualification first has certainly much to commend it. As is only natural in a first edition, many entries consist of the barest details, owing to the non-return of the publisher's circulars by members of the profession. The local directory comprises the second part of the volume, and in this the towns are arranged alphabetically. This medical directory, at the price of 7s. 6d., should find a ready sale among those who have up to now refrained from buying such a volume on the score of expense, and we have little doubt that it has come to stay.

**Forthcoming Publications.**

Messrs. J. and A. Churchill announce that Mr. F. Swinford Edwards is now engaged upon the third edition of "Diseases of the Rectum and Anus," and that a new work on "Hernia" will shortly appear from the pen of Mr. R. W. Murray. The same firm also promise new editions of Dr. Haig's "Uric Acid as a Factor in the Causation of Disease," and Mr. E. W. Lucas's "Practical Pharmacy."

### THE GRADUATES' MEDICAL SCHOOLS.

### Diary for the week March 22 to 29.

**ROYAL COLLEGE OF PHYSICIANS, Pall Mall East.**

**At 5 p.m.** Gulstonian Lectures (*continued*).

**March 24,** Dr. Herbert French, **The Influence of Pregnancy on certain Medical Diseases, and of certain Medical Diseases on Pregnancy.**

Lumleian Lectures—

**March 26,** Sir James Sawyer, M.D., **Points of Practice in Maladies of the Heart.**

**MEDICAL GRADUATES' COLLEGE AND POLYCLINIC, Chenies Street, W.C.**

**At 5.15 p.m.**

**March 23,** Mr. Jackson Clarke, **Sprains.**

**March 24,** Dr. G. F. Still, **Infantile Marasmus.**

**March 25,** Dr. E. F. Buzzard, **Compression Paraplegia.**

**March 26,** Mr. H. S. Clogg, **Peri-colic Inflammation.**

**NORTH-EAST LONDON POST-GRADUATE COLLEGE, Prince of Wales's General Hospital, Tottenham, N.**

**At 4.30 p.m.**

**March 26,** Mr. A. de Prenderville, **Some Recent Advances in Anæsthetic Technique.**

**THE POST-GRADUATE COLLEGE, West London Hospital, Hammersmith, W.**

**At noon.**

**March 23,** Dr. Low, **Pathological Demonstration.**
**March 24 and 26,** Dr. Pritchard, **Practical Medicine.**

**At 5 p.m.**

**March 24,** Dr. Robert Jones, **The Legal Aspects of Insanity in so far as these affect the Medical Man.**
**March 27,** Mr Bidwell, **Clinical—II.**

**NATIONAL HOSPITAL FOR THE PARALYSED AND EPILEPTIC, Queen Square, Bloomsbury, W.C.**

**At 3.30 p.m.**

**March 24,** Dr. F. Buzzard, **Cerebral Tumours.**

**LONDON SCHOOL OF CLINICAL MEDICINE, Seamen's Hospital, Greenwich.**

**At 2.15 p.m.**

**March 23,** Sir Dyce Duckworth, **On the Employment of Aperient Remedies.**

**3.15 p.m.**

**March 26,** Sir Wm. Bennett, **Some Clinical Aspects of the Disappearance of Pain.**

**THE HOSPITAL FOR SICK CHILDREN, Great Ormond Street, W.C.**

**At 4 p.m.**

**March 26,** Dr. Thursfield, **Leukæmia in Children.**

**CENTRAL LONDON THROAT and EAR HOSPITAL, Gray's Inn Road, W.C.**

**At 4 p.m.**

**March 26,** Dr. McKenzie, **The Deflected Nasal Septum.**

**HOSPITAL FOR CONSUMPTION AND DISEASES OF THE CHEST, Brompton, S.W.**

**At 4 p.m.**

**March 25,** Dr. Latham, **Selected Cases.**

The rate of annual subscriptions to The Hospital, either direct from the Office or through agents, is :—

For the United Kingdom... ... ... 15s. a year
To the Colonies and Abroad ... ... 17s. a year inclusive of postage in each case.

